# Cultural Representations of Borderline Personality Disorder

**DOI:** 10.3389/fsoc.2022.832497

**Published:** 2022-03-25

**Authors:** Katherine A. Munson, Carol A. Janney, Kelsie Goodwin, Madhavi Nagalla

**Affiliations:** ^1^College of Human Medicine, Michigan State University, Grand Rapids, MI, United States; ^2^Pine Rest Christian Mental Health Services, Grand Rapids, MI, United States; ^3^School of Osteopathic Medicine, A. T. Still University, Kirksville, MO, United States

**Keywords:** borderline personality disorder, culture, self-harm, non-suicidal self-injury, stressors, parasuicide

## Abstract

**Background/Objective:**

Borderline Personality Disorder (BPD) is characterized by unstable interpersonal relationships, impulsivity, and self-harm. There are many distinct stressors that predispose individuals to develop BPD or engage in self-harm behaviors. The objective of this systematic review was to compare methods of self-harm and psychological stressors in BPD across different cultures.

**Methods:**

A PubMed database search was conducted with the goal of capturing all articles (*n* = 22) that discussed methods of self-harm in BPD in any culture. Data extracted from the articles included methods of self-harm, psychological stressors, sample size, rurality, geographical location, and proportion of males to females.

**Results:**

Key differences were noted in the methods of self-harm. Eastern nations (*n* = 5) reported higher rates of self-poisoning (60%) than Western nations (11%). Western nations (*n* = 9) reported higher rates of skin-mutilating behavior (100%) than Eastern nations (80%). Two of the articles included participants from rural settings, one in the Sundarban region of India and the other in Mississippi. Notably, the Sundarban region reported the highest rate of poisoning (93%) whereas the Mississippi region reported high rates of skin mutilation. Differences were also noted in psychological stressors as the rates of interpersonal problems were higher in Western than in Eastern nations.

**Conclusions:**

Additional research should be conducted into the presentation of BPD in different cultures. An improved understanding of the cultural presentations of BPD could improve diagnosis and treatment in various populations.

## Introduction

According to the Fifth Edition of the Diagnostic and Statistical Manual of Mental Disorders (DSM-5), Borderline Personality Disorder (BPD) is characterized by “a pervasive pattern of instability of interpersonal relationships, self-image, affects, and marked impulsivity” (Chowdhury et al., 2013). Patients with BPD have a history of unstable and intense relationships, along with an extreme fear of abandonment, and they often engage in self-injurious and/or self-mutilating behaviors to seek attention from others which may or may not include suicidal intent (Chowdhury et al., 2013). Occasionally patients will accidentally complete suicide making it very difficult to discern whether suicidal intent occurred prior to the incident. It is important to classify self-harm behavior on the basis of the intent to die because psychopathological characteristics differ between the two (Del Bello et al., 2015). For instance, patients who perform non-suicidal self-injury are more likely to be women and diagnosed with BPD. The prevalence of BPD in the general population is about 1% compared to 10–12% and 20–22% in outpatient psychiatric clinics and inpatient clinics, respectively (Ellison et al., 2018). This prevalence estimate has been determined by research in primarily Western countries whereas little research has been conducted on BPD in other cultures (Ellison et al., 2018).

The few studies that have investigated the presentation of BPD in non-Western cultures have found that the manifestations of BPD are varied between cultures, which is not accounted for in the DSM-5 diagnosis for BPD. For example, Ronningstam et al. (2018) found that the occurrence of aggressive behaviors is heightened in Iranian society, meaning an Iranian patient with BPD may be more likely to demonstrate aggression. Additionally, Italian patients with BPD show less impulsivity and parasuicidal acts, which may be linked to the Italian culture which is predominantly collectivistic and Catholic (Ronningstam et al., 2018). Collectivistic and family-oriented culture may prevent Italian individuals from exhibiting the classic impulsive and suicidal behaviors of BPD. Thus, it is possible that BPD can go misdiagnosed or undiagnosed when failing to account for a patient's cultural context.

Because there has been minimal research on BPD in non-Western cultures, there is a debate on whether or not BPD is solely a “Western” disorder with the development of BPD prevented in traditional and collectivistic societies of non-Western cultures. In attempt to study this hypothesis, suicide attempters from a Mumbai, India hospital were studied for the presence of BPD (Pinto et al., 2000). Using the DSM-IV criteria, Pinto and associates found that 17.3% (*n* = 13) of suicide attempters had a diagnosis of BPD, suggesting that BPD does exist in India and may go underreported. However, the study was conducted at a large urban hospital which may be more “westernized” than small, rural communities. There is still an incomplete understanding on whether BPD goes undiagnosed in non-Western cultures or that it simply does not exist in these societies.

Additionally, some studies use different terminology for BPD such as “emotionally unstable” or “impulsive” which may indicate different BPD pathology depending on the culture (Nath et al., 2008). China has not included BPD in the Third Edition of the Chinese Classification of Mental Disorders (CCMD-3), but does include “impulsive personality disorder” which heavily overlaps with BPD (Wang et al., 2012). There are certain behaviors that define BPD that may not be suitable for Chinese culture, such as reckless driving, promiscuous sex, and substance abuse (Wang et al., 2012). Owning a car is uncommon in China, sex is a taboo topic in Chinese culture, and many illicit drugs are heavily controlled in China (Wang et al., 2012). Therefore, certain behaviors associated with BPD may not be appropriate for the CCMD-3. This begs the question: can the diagnosis of BPD be used for all cultures or is it primarily Western-driven?

The primary purpose of this paper is to investigate BPD behaviors across cultures. This report will focus on non-suicidal self-injury or “parasuicide” which includes any self-mutilating behavior without the explicit intent to die by suicide. Many distinct psychological stressors predispose individuals to BPD, such as interpersonal or family issues. This report will investigate cultural differences in these stressors. To our knowledge, there has been no analysis of cultural differences in BPD.

This literature review will address the following questions:

1. How does non-suicidal self-harm or BPD behaviors differ across cultures?
2. How do psychological stressors of BPD vary across cultures?

## Methods

**Figure 1:**
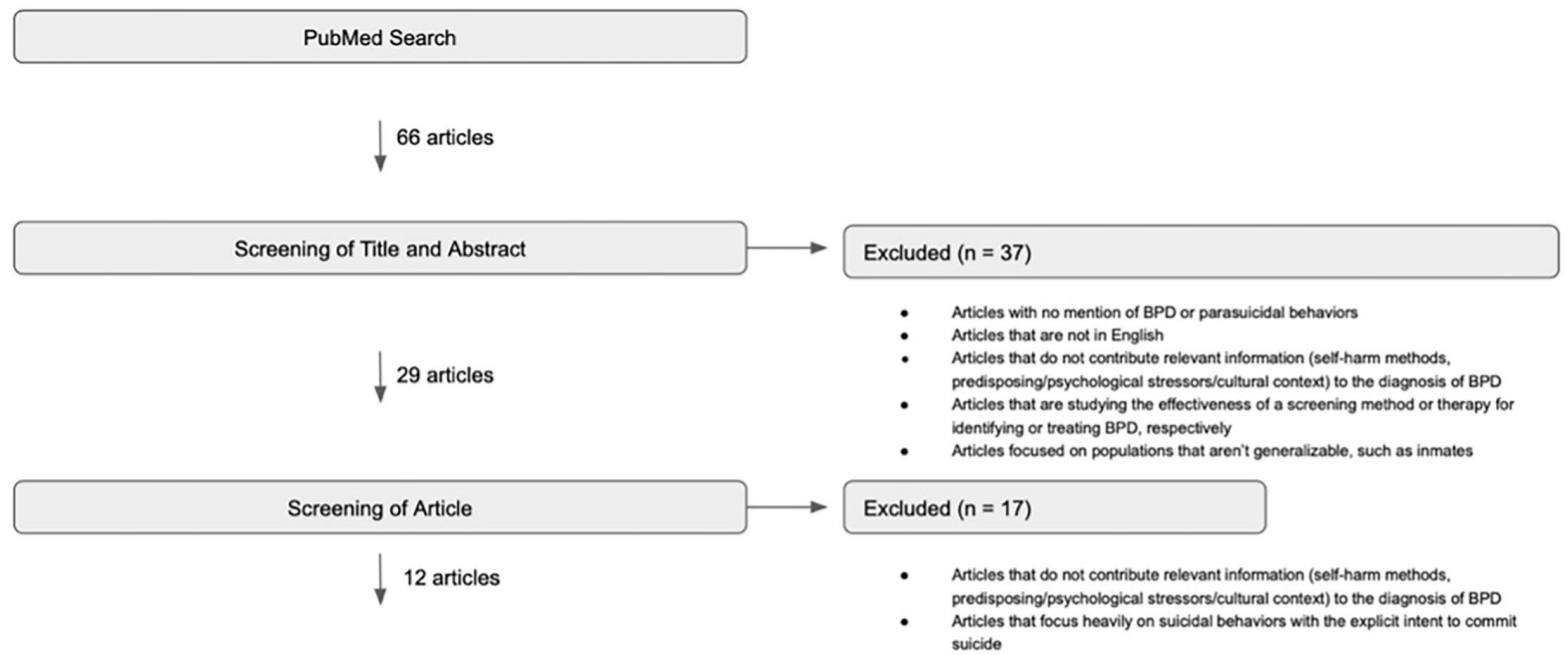
PubMed search for articles pertaining to Borderline Personality Disorder presentations (3/2020).

A literature search was conducted using PubMed to identify articles that discuss various cultural presentations of Borderline Personality Disorder. The keywords were established with the assistance of an experienced librarian (see Table 1). Additionally, non-exhaustive initial searches and reference/citation lists were explored to identify important keywords and to find articles pertaining to BPD not captured in the main search. The details of these searches may be found in the Appendix. The results of the PubMed search were screened according to the inclusion and exclusion criteria defined in Table 2. Articles were included if they discussed BPD and parasuicidal behaviors in a specific culture or discussed various clinical manifestations of BPD from a cultural context. Articles were excluded if they did not mention BPD and parasuicidal behaviors in a specific culture, were not in English, did not contribute relevant information to the review, studied a screening method or therapy for BPD, focused on non-generalizable populations, or focused heavily on suicidal behaviors. The initial 66 articles were screened by title and abstract using these criteria, resulting in the exclusion of 37 articles (Figure 1). The full texts of the remaining 29 articles were screened using Table 2 criteria, resulting in the exclusion of an additional 17 articles. Thus, this literature search identified 12 published articles pertaining to BPD presentations, in addition to 5 articles from initial searches and 4 from reference/citation lists. Finally, 1 additional article was discovered by a reviewer giving us a total of 22 articles for review.

**Table 1:** Literature search keywords.

	**AND**	**AND**
*borderline[tiab]* OR “*borderline disorder”[tiab]* OR “*borderline personality disorder”[tiab]*	suicide[ti] AND (pattern*[ti] OR behavior[ti]) OR “parasuicidal”[ti] OR “self harm”[ti] OR “self injurious behaviors”[ti] OR “suicidal behavior*”[ti] OR “suicidal behavior*”[ti] OR “suicide ideation”[ti] OR “suicidal ideation”[ti]	“North America” OR Canada OR Africa OR Asia OR India OR Europe OR China OR Japan OR Korea OR Taiwan OR Russia OR “South America” OR Eastern OR western OR race OR ethnicity OR origin OR “geographic region” OR “countries of origin” OR “country of origin” OR immigrant* OR refugee* OR Indigenous OR “First Nations” OR “Alaska Native” OR “Native American” OR latin* OR Hispanic OR “African American” OR black

**Table 2:** Inclusion/exclusion criteria used to screen the results of the PubMed search.

**Inclusion criteria**
**Any article that mentions BPD and parasuicidal behaviors in a specific culture or in multiple different cultures**
**Articles that discuss the various clinical manifestations of BPD and the socio-cultural context of personality disorders**
**Articles are not limited to any particular geographic location**
**Exclusion criteria**
**Articles with no mention of BPD and parasuicidal behaviors**
**Articles that are not in English**
**Articles that do not contribute relevant Information (self-harm methods, psychological stressors, cultural context) to the diagnosis of BPD**
**Articles that are studying the effectiveness of a screening method for identifying BPD**
**Articles that are studying the effectiveness of a therapy for treating BPD**
**Articles focused on non-generalizable populations, such as inmates**
**Articles that focus heavily on suicidal behaviors with the explicit intent to die by suicide**


Data extracted from the articles included methods of self-harm, psychological stressors, sample size, rurality, geographical location, and proportion of males to females. Some articles explicitly attributed particular self-harm methods and psychological stressors to people with a diagnosis of BPD whereas others did not make this distinction between BPD and other psychiatric disorders. The articles that did not make this distinction were still included because some cultures may underdiagnose BPD or classify it as a different disorder (Pinto et al., 2000).

The articles were divided into the subgroups Eastern and Western nations. Eastern nations included East Asia, East India/Morocco, Hong Kong, India, and Japan. Western nations included Canada, Croatia, Italy, Norway, Sweden, United States, and Western Australia. While this classification system results in a broad generalization of multiple unique cultures into only two categories, it highlights the need for more data on BPD from a global perspective. There are vast cultural differences within each of these categories, thus this analysis may not represent the complexities of BPD presentation in multiple distinctive cultures. Rurality was defined by each article. Some articles defined their study population as “rural,” “urban,” or “mixed rural/urban” which is indicated in Tables 3, 4.

**Table 3:** Methods of self-harm by study based on 13 BPD articles.

	**Publication** **year**	**Sample size** **(*n*)**	**Rurality**	**Population**	**BPD mentioned clearly with self-harm**	**Skin mutilation %**	**Burning** **%**	**Poisoning %**	**Overdose** **%**	**Impact** **%**	**Asphyxiation** **%**
**Hong Kong** Prevalence of borderline personality disorder and its clinical correlates in Chinese patients with recent deliberate self-harm	2010	30	Urban	Chinese	X^a^	20	3	3	87	3	
**India (Sundarban Region)** Sociocultural Context of Suicidal Behavior in the Sundarban Region of India	2013	89	Rural	Indian		^b^		93	2		5
**Japan (Tokyo)** Clinical characteristics of suicidal behavior in an intensive care unit at a university hospital in Japan: A 7-year observational study	2018	971	Urban	Japanese		9	4	4	75	5	3
**Japan (Tokyo)** Motivation factors for suicidal behavior and their clinical relevance in admitted psychiatric patients	2017	155	Urban	Japanese		40			30	20	14
**Croatia (Osijek)** Self-injury in adolescents: A five-year study of characteristics and trends	2019	105	Urban	Not specified		92	4			4	
**Italy (Rome)** Deliberate self-harm behavior among Italian young adults: correlations with clinical and nonclinical dimensions of personality	2012	365	Urban	Italian		65	7			11	
**Italy (Naples)** Prevalence and clinical correlates of deliberate self-harm among a community sample of Italian adolescents	2011	234	Urban	Italian		65	6			12	
**Norway (West Coast)** Desire to survive emotional pain related to self-harm: A Norwegian hermeneutic study	2010	13	—	Not specified	X	✓^c^	✓		✓		
**Sweden (Umeå)** Personality disorders in parasuicide	2001	35	Urban	Not specified	X	✓		✓	✓	✓	✓
**United States (Rhode Island)** Nonsuicidal self-injury and suicide: Differences between those with and without borderline personality disorder	2020	389	Urban	87.2% Caucasian, 4.1% African American, 2.8% Hispanic, 1.0% Asian, 3.3% Portuguese, 0.1% American Indian, 1.5% Other	X	95	5			26	
**United States (Mississippi)** Deliberate self-harm among underserved adolescents: the moderating roles of gender, race, and school-level and association with borderline personality features	2011	1931	Rural	67.0% Caucasian		60	15			46	
				33.0% African American		77	21			58	
**United States (New York)** Psychiatric impairment among adolescents engaging in different types of deliberate self-harm	2008	30	Urban	69.0% Hispanic, 20.0% African American, 4.0% Caucasian, 4.0% Other		74	10		33		3
**Western Australia** Risk factors for repetition of a deliberate self-harm episode within seven days in adolescents and young adults: A population-level record linkage study in Western Australia	2016	16966	Mix (mostly urban)	93.3% Non-Aboriginal, 6.7% Aboriginal		✓			45		

a *X Signifies that the article clearly attributes the methods of self-harm to BPD patients*.

b *Blank squares signify no data*.

c *✓ Signifies that the article mentioned this method of self-harm but did not provide a percentage*.

**Table 4:** Psychological stressors by study based on 12 BPD articles.

	**Publication year**	**Sample size (*n*)**	**Rurality**	**Population**	**BPD mentioned clearly with psychological** **stressors**	**Interpersonal problems %**	**Family issues %**	**Financial problems %**	**Health problems** **%**	**Abuse** **%**	**Violence/** **accident** **%**	**Miscarriage/** **abortion** **%**	**Examination** **failure** **%**	**Moving %**	**Mental shock %**	**Imprisoned Relatives/** **Personally Imprisoned %**
**Hong Kong** Prevalence of borderline personality disorder and its clinical correlates in Chinese patients with recent deliberate self-harm	2010	30	Urban	Chinese	X^a^	^b^				63						
**East Asia** Cultural aspects in symptomatology, assessment, and treatment of personality disorders	2018	—	—	East Asian	X	✓^c^										
**East India/Morocco** Cultural factors in the emergence of borderline pathology	1996	2	—	East Indian/Moroccan		50	50					50				
**India (Mumbai)** Borderline personality disorder exists in India	2000	75	Urban	Indian						62						
**India (Sundarban Region)** Sociocultural Context of Suicidal Behavior in the Sundarban Region of India	2013	89	Rural	Indian		12	88	9	3	19			3		4	
**Japan (Tokyo)** Clinical characteristics of suicidal behavior in an intensive care unit at a university hospital in Japan: A 7-year observational study	2018	971	Urban	Japanese		24	33	18	6							
**Japan (Tokyo)** Motivation factors for suicidal behavior and their clinical relevance in admitted psychiatric patients	2017	155	Urban	Japanese		✓										
**Canada (Manitoba)** A comparison of self-harm presentations to emergency services: Nonsuicidal self-injury versus suicide attempts	2020	158	—	Not specified		35										
**Croatia (Osijek)** Self-injury in adolescents: A five-year study of characteristics and trends	2019	105	Urban	Not specified		39	13		3	19	5	5		16		
**Italy** Cultural aspects in symptomatology, assessment, and treatment of personality disorders	2018	—	—	Italian	X		✓									
**Italy (Naples)** Prevalence and clinical correlates of deliberate self-harm among a community sample of Italian adolescents	2011	234	Urban	Italian		61		12	49	50	Y^d^	1				30
**Norway (West Coast)** Desire to survive emotional pain related to self-harm: A Norwegian hermeneutic study	2010	13	—	Not specified	X					Y^d^	46					

a *X Signifies that the article clearly attributes the psychological stressors to BPD patients*.

b *Blank squares signify no data*.

c *✓ Signifies that the article mentioned this psychological stressor but did not provide a percentage*.

d *Y signifies that the article reported data on specific subcategories of violence or abuse but did not provide data on the total number experiencing violence or abuse*.

## Results

### Literature Search

The initial PubMed search performed in March 2020 yielded 66 results. After a screening of titles and abstracts, 37 articles were excluded because they did not mention BPD and parasuicidal behaviors, were not in English, did not contribute relevant information to the objectives of this analysis, or focused on a non-generalizable population. After screening the full text of the remaining 29 articles, 17 were excluded because they did not contribute relevant information to the objectives of this literature review or they focused very heavily on suicidal behaviors with the explicit intent to die by suicide. A repeat literature search with the same keywords was conducted in January 2022 which yielded 72 total results, 66 articles from our previous search and 6 new articles. None of the 6 articles were included for the following reasons: focused very heavily on suicidal behaviors, focused on non-generalizable populations, or did not contribute relevant information to this study. The details of the PubMed search performed in March 2020 can be found in Figure 1.

Using a reference and citation search from the 12 articles (Jacobson et al., 2008; Nath et al., 2008; Holm and Severinsson, 2010; Wong et al., 2010; Cerutti et al., 2011, 2012; Gratz et al., 2012; Del Bello et al., 2015; Hu et al., 2016; Hayashi et al., 2017; Takeuchi et al., 2018; Bježančević et al., 2019) identified through the PubMed search, an additional 4 articles (Paris, 1996; Pinto et al., 2000; Söderberg, 2001; Wang et al., 2012) were identified. Five other articles (Bježančević et al., 2019; Ronningstam et al., 2018; Haliczer et al., 2019; Chartrand et al., 2020; Levine et al., 2020) included in the literature review are from non-exhaustive initial searches that were conducted while refining the keywords for the main search. One additional article was discovered by a reviewer and included in the review (You et al., 2012). Overall, a total of 22 articles were included in the literature review, 5 from the non-exhaustive initial searches, 12 from the main search, 4 from the reference and citation search, and 1 from the reviewer. Eighteen of the 22 articles directly contributed data to the self-harm methods and psychological stressors (Tables 3, 4). From the remaining 4 articles (Nath et al., 2008; Wang et al., 2012; Del Bello et al., 2015; Haliczer et al., 2019), relevant information was extracted and incorporated into the introduction and discussion sections because they provided cultural context on BPD. Of the 22 articles reviewed, 14 (64%) mentioned methods of self-harm and 11 (50%) mentioned psychological stressors within the context of BPD.

The articles that clearly defined self-harm methods and psychological stressors in participants with BPD can be identified in Tables 3, 4 with a check mark in the column “BPD mentioned clearly with self-harm/psychological stressors.” Seven of the 22 articles (32%) clearly attributed self-harm methods/psychological stressors to a BPD diagnosis. All of the articles besides one (95%) used a diagnosis of BPD. The exception was conducted in Kolkata, India and used the diagnosis of emotionally unstable personality disorder which they defined as impulsive, borderline or both (Nath et al., 2008).

### Methods of Self-Harm

Fourteen (64%) studies mentioned methods of self-harm that were seen in a particular culture (Söderberg, 2001; Jacobson et al., 2008; Holm and Severinsson, 2010; Wong et al., 2010; Cerutti et al., 2011; Gratz et al., 2012; You et al., 2012; Hu et al., 2016; Hayashi et al., 2017; Takeuchi et al., 2018; Bježančević et al., 2019; Chartrand et al., 2020; Levine et al., 2020). The total sample size for these studies is 26,095, with 63% (*n* = 16,313) women and 37% (*n* = 9,782) men. The methods of self-harm reported in each article were summarized into the following categories: skin mutilation, burning, poisoning, overdose, impact, and asphyxiation which is detailed in Table 5.

**Table 5:** Self-harm methods for BPD which were indicated in the literature.

**Skin mutilation**	**Cutting skin (Jacobson et al., 2008; Holm and Severinsson, 2010; Wong et al., 2010; Cerutti et al., 2011; Gratz et al., 2012; You et al., 2012; Hu et al., 2016; Takeuchi et al., 2018; Bježančević et al., 2019; Levine et al., 2020), wrist/arm cutting (Söderberg, 2001; Hayashi et al., 2017), cutting other parts of body (Hayashi et al., 2017), carving words into skin (Cerutti et al., 2011; Bježančević et al., 2019; Levine et al., 2020), carving pictures into skin (Cerutti et al., 2011; Bježančević et al., 2019), scratching skin (Cerutti et al., 2011; Gratz et al., 2012; Bježančević et al., 2019; Levine et al., 2020), picking skin (Levine et al., 2020), wound picking (Levine et al., 2020), interference with wound healing (Cerutti et al., 2011; Bježančević et al., 2019), stab/puncture (Jacobson et al., 2008), biting (Cerutti et al., 2011; Gratz et al., 2012; You et al., 2012; Bježančević et al., 2019), chewing mouth (Levine et al., 2020), sticking pins, needles, staples, into skin (Cerutti et al., 2011; Bježančević et al., 2019), rubbing glass into skin (Cerutti et al., 2011; Bježančević et al., 2019), using bleach/oven cleaner to scrub skin (Cerutti et al., 2011; Bježančević et al., 2019), rubbing sandpaper on skin (Cerutti et al., 2011; Bježančević et al., 2019), genital injury/mutilation (Levine et al., 2020), hurt self while masturbating (Levine et al., 2020), and hair pulling (Levine et al., 2020)**
**Burning**	**Burning with cigarettes (Cerutti et al., 2011; Bježančević et al., 2019), burning with lighter/match (Cerutti et al., 2011; Bježančević et al., 2019), burning skin (unspecified) (Söderberg, 2001; Jacobson et al., 2008; Holm and Severinsson, 2010; Gratz et al., 2012; You et al., 2012; Bježančević et al., 2019; Levine et al., 2020), charcoal burning/gas poisoning (Wong et al., 2010), and use of charcoal briquettes (Takeuchi et al., 2018)**
**Poisoning**	**Corrosives ingestion (Wong et al., 2010), chemical poisoning (Takeuchi et al., 2018), Carbon monoxide intoxication (Söderberg, 2001; Takeuchi et al., 2018), poisoning with agrochemical pesticides (Chartrand et al., 2020), poisoning with household chemicals (Chartrand et al., 2020), and poisoning with indigenous poisons (Chartrand et al., 2020)**
**Overdose**	**Poisoning with non-illicit drug (Hu et al., 2016), poisoning with illicit drug (Hu et al., 2016), poisoning with medicines (Chartrand et al., 2020), overdosing on prescribed psychotropics (Hayashi et al., 2017), overdosing on other prescribed medicine (Hayashi et al., 2017), overdosing on OTC medicine (Hayashi et al., 2017), drug overdose (Söderberg, 2001; Jacobson et al., 2008; Holm and Severinsson, 2010; Wong et al., 2010; Takeuchi et al., 2018), and alcohol overdose (Holm and Severinsson, 2010)**
**Impact**	**Traffic (Hayashi et al., 2017; Takeuchi et al., 2018), banging (Gratz et al., 2012), banging head (Cerutti et al., 2011; You et al., 2012; Bježančević et al., 2019), banging head, arms, and legs (Levine et al., 2020), stabbing items in the body (Bježančević et al., 2019), hitting self (Levine et al., 2020), hitting body on a subject (Bježančević et al., 2019), punching self (Cerutti et al., 2011; Gratz et al., 2012; You et al., 2012; Bježančević et al., 2019), and jumping from height (Söderberg, 2001; Wong et al., 2010; Hayashi et al., 2017; Takeuchi et al., 2018)**
**Asphyxiation**	**Drowning (Takeuchi et al., 2018) and strangulation (Söderberg, 2001; Jacobson et al., 2008; Hayashi et al., 2017; Takeuchi et al., 2018; Chartrand et al., 2020)**

Out of the 14 self-harm studies, 93% (*n* = 13) reported skin mutilation, 71% (*n* = 10) reported burning, 71% (*n* = 10) reported impact, 62% (*n* = 8) reported overdose, 38% (*n* = 5) reported asphyxiation, and 31% (*n* = 4) reported poisoning. There were notable differences in the methods of self-harm between Eastern and Western nations which can be found in Table 6. In Eastern nations, the predominant forms of self-harm were overdose and poisoning. Wong et al. (Hong Kong) reported 87% (*n* = 26) of participants with BPD overdosed, Takeuchi et al. (Tokyo, Japan) reported 75% (*n* = 732) of participants overdosed, and Chowdhury et al. (Sundarban, India) reported 93% (*n* = 83) of participants self-poisoned. The predominant form of self-harm in Western nations was skin mutilation which was seen in 92% (*n* = 97) of participants from Croatia (Bježančević et al., 2019), 95% (*n* = 431) of participants from Rhode Island (Levine et al., 2020), and 77% (*n* = 1,487) for the African American population from Mississippi (Gratz et al., 2012).

**Table 6:** Comparison of methods of self-harm between Eastern and Western nations.

**Method of self-harm**	**Eastern nations (*n* = 5^a^)**	**Western nations (*n* = 9^a^)**
Skin mutilation	80% (*n* = 4)	100% (*n* = 9)
Burning	60% (*n* = 3)	78% (*n* = 7)
Poisoning	75% (*n* = 3)	11% (*n* = 1)
Overdose	100% (*n* = 4)	44% (*n* = 4)
Impact	80% (*n* = 4)	67% (*n* = 6)
Asphyxiation	75% (*n* = 3)	22% (*n* = 2)

a *Number of journal articles*.

Two of the articles included participants from rural settings, one in the Sundarban region of India (Chartrand et al., 2020) and the other in Mississippi (Gratz et al., 2012). Notably, the Sundarban region reported the highest rate of poisoning (93%) which included agrochemical pesticides, household chemicals, and indigenous poisons. Additionally, the Sundarban article was the only article which did not mention skin mutilation as a method of self-harm. The rural Mississippi article showed high rates of skin mutilation, 77% for African Americans (*n* = 1,487) and 60% for Caucasians (*n* = 1,159), and impact, 58% for African Americans (*n* = 1,120) and 46% for Caucasians (*n* = 888).

### Psychological Stressors

Eleven (52%) studies mentioned psychological stressors that predisposed participants to perform self-harm (Paris, 1996; Pinto et al., 2000; Holm and Severinsson, 2010; Wong et al., 2010; Cerutti et al., 2012; Hayashi et al., 2017; Ronningstam et al., 2018; Takeuchi et al., 2018; Bježančević et al., 2019; Chartrand et al., 2020). The total sample size for these studies is 1,832, with 66% (*n* = 1,209) women and 34% (*n* = 623) men. The psychological stressors reported were grouped into the following categories: interpersonal problems, family issues, financial problems, health problems, abuse, violence/accident, miscarriage/abortion, examination failure, moving, mental shock, and imprisoned relatives/personally imprisoned (Table 7).

**Table 7:** Psychological stressors for BPD which were indicated in the literature.

**Interpersonal problems (Hayashi et al., 2017; Ronningstam et al., 2018; Takeuchi et al., 2018)**	**End of a dating relationship (Bježančević et al., 2019), broken love affair (Paris, 1996; Chartrand et al., 2020), death/loss of a close person (Bježančević et al., 2019), social shame (Chartrand et al., 2020), low social support (Cerutti et al., 2012), and threat of rejection (Chartrand et al., 2020)**
**Family issues (Paris, 1996; Ronningstam et al., 2018; Takeuchi et al., 2018)**	**Marital conflict (Chartrand et al., 2020), conflict with parents/guardians (Chartrand et al., 2020), conflict with in-laws (Chartrand et al., 2020), parents' divorce (Bježančević et al., 2019), and family discord (Chartrand et al., 2020)**
**Financial problems (Takeuchi et al., 2018; Bježančević et al., 2019)**	**Economic distress (Chartrand et al., 2020), dowry conflict (Chartrand et al., 2020), and occupational problems (Takeuchi et al., 2018)**
**Health problems (Takeuchi et al., 2018; Bježančević et al., 2019)**	**Chronic illness (Chartrand et al., 2020), disease in family (American Psychiatric Association, 2013), and providing care for someone ill (Bježančević et al., 2019)**
**Abuse**	**Sexual abuse (Holm and Severinsson, 2010; Wong et al., 2010; American Psychiatric Association, 2013; Bježančević et al., 2019), physical abuse (Holm and Severinsson, 2010; Wong et al., 2010; Bježančević et al., 2019), childhood sexual/physical abuse (Pinto et al., 2000), emotional abuse (Holm and Severinsson, 2010), psychological maltreatment (Bježančević et al., 2019), neglected (Holm and Severinsson, 2010; Bježančević et al., 2019), rape (Bježančević et al., 2019), and domestic violence (Chartrand et al., 2020)**
**Violence/accident**	**Peer violence (Bježančević et al., 2019), witnessed violence (Holm and Severinsson, 2010), witnessed family violence (Bježančević et al., 2019), injured in a serious accident (Bježančević et al., 2019), witnessing a serious accident (Bježančević et al., 2019), natural disasters (Bježančević et al., 2019), witnessed assault/robbery (Bježančević et al., 2019), and robbed/physically assaulted (Bježančević et al., 2019)**
**Miscarriage/abortion**	**Miscarriage (Bježančević et al., 2019), abortion (Paris, 1996), and spontaneous/deliberate termination of pregnancy (Bježančević et al., 2019)**
**Other**	**Examination failure (Chartrand et al., 2020), moving (Bježančević et al., 2019), mental shock (Chartrand et al., 2020), and imprisoned relatives/personally imprisoned (Bježančević et al., 2019)**

Out of the 11 studies that mentioned psychological stressors, 67% (*n* = 8) reported interpersonal problems, 50% (*n* = 6) reported abuse, 42% (*n* = 5) reported family issues, 33% (*n* = 4) reported health problems, 25% (*n* = 3) reported financial problems, 25% (*n* = 3) reported violence/accident, and 8% (*n* = 1) reported examination failure, moving, miscarriage, and abortion each. There were notable differences in psychological stressors between Eastern and Western nations which can be found in Table 8.

**Table 8:** Comparison of psychological stressors between Eastern and Western nations.

**Psychological stressors**	**Eastern nations (*n* = 7^a^)**	**Western nations (*n* = 5^a^)**
Interpersonal problems	71% (*n* = 5)	60% (*n* = 3)
Family issues	43% (*n* = 3)	40% (*n* = 2)
Financial problems	29% (*n* = 2)	20% (*n* = 1)
Health problems	29% (*n* = 2)	40% (*n* = 2)
Abuse	43% (*n* = 3)	60% (*n* = 3)
Violence/accident	0% (*n* = 0)	60% (*n* = 3)

a *Number of journal articles*.

One of the articles (Chartrand et al., 2020) included participants from a rural region, the Sundarban region of India. Notably, this article had the highest rate of family issues, with 88% (*n* = 74) of participants reporting a family issue such as marital conflict, conflict with parents/guardians, and/or conflict with in-laws. Several of the articles reported interpersonal problems, but the rates were higher in Western than in Eastern nations. For rates of interpersonal problems, Chartrand et al. (Manitoba, Canada) reported 35% (*n* = 55), BjeŽančević et al. (Osijek, Croatia) reported 39% (*n* = 41), and Cerutti et al. (Naples, Italy) reported 61% (*n* = 143). In comparison, Eastern nations reported lower rates for interpersonal problems such as 12% (*n* = 11) in the Sundarban region of India (Chartrand et al., 2020) and 24% (*n* = 233) in Tokyo, Japan (Takeuchi et al., 2018).

## Discussion

This literature review has identified notable cultural differences in self-harm and psychological stressors for patients with BPD. These findings warrant further study into the cultural presentations of BPD.

Based on the studies reviewed, self-poisoning and overdose are more commonly seen in Eastern vs. Western nations. All of the articles that were conducted in Eastern nations (Hong Kong, India, Japan) (Wong et al., 2010; Hayashi et al., 2017; Takeuchi et al., 2018; Chartrand et al., 2020) reported self-poisoning or overdose at higher rates than Western nations. Ronningstam et al. and Haliczer et al. reported that East Asians are more likely to engage in suppression of emotions and attenuated behavioral reactivity to emotional stimuli compared to European Americans. Additionally, traditional Chinese culture, which is heavily influenced by Confucian philosophy, encourages group cohesion, collectivism, self-control, and stoicism. Chinese culture typically discourages impulsive actions and overtly expressing one's emotions (Wong et al., 2010). Also, self-poisoning may be more common in Eastern nations because poisons/chemicals may be more readily accessible in these rural regions. Chowdhury et al. reported that the most common method of self-harm among a sample of people from the rural Sundarban region of India was poisoning with agrochemical pesticides. In this Eastern region of the world, agrochemical pesticides may be more accessible than other modes of self-harm.

Another possible explanation for the differences in self-poisoning and overdose rates between Eastern vs. Western countries may be the patient populations. Most of the studies in Eastern nations identified patients that were admitted to tertiary care facilities such as ICUs, psychiatric hospitals, or emergency rooms. In contrast, the Western studies included patients from outpatient as well as inpatient settings (emergency rooms, inpatient psychiatric hospitals, outpatient psychiatry programs, and schools). Feasibly, more life-threatening methods of self-harm, such as poisoning, were detected in Eastern nations. This finding points to the need for more school and community-based research in Eastern nations, which may help detect minor methods of self-harm, such as cutting and scratching the skin. Supporting this hypothesis is one study conducted by You et al. that interviewed Chinese adolescents from six secondary schools in Hong Kong about BPD symptoms (You et al., 2012). Adolescents reported the following methods of self- harm: cutting, burning, biting, punching, and banging of the head. This article demonstrates that minor methods of self-harm may be present in non-Western societies but have not been well studied.

In contrast, skin mutilation is a more common method of self-harm in Western vs. Eastern nations. All of the Western articles (conducted in Croatia, Italy, Norway, Sweden, United States, and Western Australia) reported skin mutilation at higher rates than Eastern nations. Methods of skin mutilation, such as cutting or carving skin, are very expressive methods of self-harm which fits the concept that people from Western nations tend to be more emotionally expressive.

There is debate on whether BPD is solely a Western disorder and more traditional societies are protected from engaging in BPD behavior. Because BPD is defined as “a pervasive pattern of instability of interpersonal relationships, self-image, affects, and marked impulsivity”, some have theorized that more collectivistic, traditional societies thwart the development of BPD pathology (Paris, 1996; Pinto et al., 2000; Ronningstam et al., 2018). Globalization has permitted the rapid transmission of ideas across cultures which may have heightened the susceptibility of non-Western nations to DSM-5 BPD pathology. Little research on the presentation of BPD has been conducted in rural societies with limited exposure to the forces of globalization.

One non-Western, rural study was identified in this review which looked at suicidal behavior in the Sundarban region of India (Chartrand et al., 2020). They interviewed participants who had engaged in suicidal behavior and asked about their intent to die. Interestingly, many said that they were “uncertain” about their intent to die because if they said yes they would face legal complications and if they said no they may face social humiliation or stigma. Deliberate self-harm without the intent to die is locally known as “Jukhimara” or “Jukhi” which means one wants to communicate his/her sufferings as an alarm or wants to achieve something (Chartrand et al., 2020). Socially, Jukhi is seen as bad character and may negatively affect a woman's marriage prospects (Chartrand et al., 2020). This cultural definition for parasuicidal behavior in a relatively remote region of the world suggests that BPD may exist without the influences of globalization. However, the presentation of BPD appears to differ in this culture as many of the participants engaged in self-poisoning but not skin mutilation, the latter being a more emotionally expressive form of self-harm. Interestingly, only 2 out of the 89 participants were diagnosed with BPD which may indicate a lack of awareness of this disorder. Additional research should be conducted to gain more insight into BPD presentations in rural settings.

The Western studies identified in this review consisted mainly of Caucasians, with little investigation into other races. One article conducted in rural Mississippi did investigate self-harm and BPD comparing African American to Caucasian youth but still had a predominantly Caucasian sample (67% Caucasian, 33% African American) (Gratz et al., 2012). In this study, African American boys reported higher rates of most self-harm behaviors than their peers. The highest rates of cutting were seen in African American boys and Caucasian girls. Interestingly, BPD features did not explain the higher rates of self-harm among African American boys indicating that the current diagnostic criteria for BPD may be non-inclusive for this population (Gratz et al., 2012). Further research should be conducted with participants of African American race to create more inclusive BPD criteria.

Based on this review, BPD presentation may differ across cultures. Hence, it is important to consider how BPD may present in immigrants. It is unknown if immigrants are more likely to demonstrate BPD manifestations of their native culture or of their host culture. Their presentation may depend on the age at which they immigrated and the amount of exposure to their native culture they have experienced while living in their host culture. Paris and associates have proposed a model of BPD pathology in immigration (Paris, 1996). Briefly, psychological risk factors which predispose individuals to BPD pathology may be present in individuals from traditional societies but social protective factors suppress the diagnosis of these traits as BPD in their native culture. Thus, the BPD pathology emerges only after immigration once one loses community support from their native country. More research should investigate how BPD pathology is affected by immigration. Research on immigration and BPD would expand our knowledge on how globalization shapes BPD pathology. It will help us better understand how sociocultural factors may be protective against BPD pathology.

## Limitations

Very few articles (*n* = 7) that were identified in this review investigated self-harm methods and psychological stressors directly in a population of people with diagnosed BPD (Söderberg, 2001; Holm and Severinsson, 2010; Wong et al., 2010; Ronningstam et al., 2018; Levine et al., 2020). Most articles included samples of participants with multiple different psychological disorders, including major depressive disorder, generalized anxiety disorder, and various personality disorders. These articles were included to expand the scope of this review and account for cultures who may underdiagnose BPD. Because many of these articles included multiple disorders, it was difficult to attribute our findings directly to BPD when they may be manifestations of another diagnosis. Additionally, BPD commonly co-occurs with mood disorders and other personality disorders, making it more difficult to identify manifestations of BPD. More research should be conducted with a focus on people with diagnosed BPD and their presentation so it can be more clearly defined. Research on BPD would improve our understanding of BPD presentation, which would help providers accurately diagnose and treat BPD.

Many of the articles included in this study attempted to determine whether or not suicidal intent was present when a self-harm action occurred, however it is very difficult to accurately discern intent. Seven of the fourteen articles that mentioned self-harm used an objective method to determine whether or not suicidal intent was present, including the Suicide Intention Scale, Deliberate Self-Harm Inventory, Lifetime Parasuicide Count, or simply asking if they had intentions to die by suicide (Jacobson et al., 2008; Wong et al., 2010; Cerutti et al., 2011; Gratz et al., 2012; Hayashi et al., 2017; Bježančević et al., 2019; Chartrand et al., 2020). The remaining articles either did not investigate intent or assumed no suicidal intent because the self-harm methods were less severe, i.e., scratching, picking, or cutting skin. Even articles that did investigate suicidal intent had difficulty truly discerning whether or not a person wanted to die by suicide. Many people will say they are uncertain and often there is a spectrum to defining intent. This review focused on self-harm without the intent to die by suicide but there is uncertainty of the intent in many articles. Therefore, our results may represent more severe methods of self-harm if articles included participants who had some intent to die.

Another limitation of this review is that the terminology used to describe BPD and its manifestations is not universal. We attempted to use a wide range of terms that encompass “self-harm” but it was difficult to capture all possibilities. There may be articles that have been missed because of varying terminology.

Our classification of countries into “Western” and “Eastern” societies was necessary to draw comparisons between different regions, but it oversimplified the cultural differences within each country. Thus, this analysis may not represent the complexities of BPD presentation in multiple distinctive cultures. This limitation highlights the need for more data on BPD from a global perspective.

This review resulted in a small sample size of articles (*n* = 22) which highlights the need for more research on this topic. All of the reviewed articles were in English, which further limits this review. Research is especially lacking in the continents of South America, Africa, and Asia. To address this need, we have developed a survey and administered it globally with the goal of expanding the literature on BPD presentation in different cultures, especially in the aforementioned regions.

## Conclusion

This literature review contributes an improved understanding of methods of self-harm and psychological stressors in BPD across cultures. Several articles were identified from various cultures, including but not limited to India, Hong Kong, Japan, Croatia, Norway, and Sweden. Poisoning and overdose were more prevalent in Eastern nations, whereas skin mutilation was more common in Western nations. We hypothesize that self-poisoning and overdose are more common in Eastern nations because they are less expressive acts than cutting or skin mutilation. Self-poisoning and overdose allow one to remove oneself from a situation and avoid engaging in emotional discourse. Future research should focus on BPD presentation in different cultures, races, and in more rural regions of the world. Further research will enhance our understanding of the pathogenesis of BPD in different cultures, thus improving diagnosis and outcomes for patients with BPD across the globe.

## Author Contributions

KAM: performed the literature review, compiled and interpreted the data, and drafted the article. CAJ: contributed to the design of the literature review, analysis of the data, and critically revised the article. KG: contributed to the literature review, compilation of data, and critical review of the article. MN: conceived the research question, contributed to the analysis of the data, and critically revised the article. All authors contributed to the article and approved the submitted version.

## Conflict of Interest

The authors declare that the research was conducted in the absence of any commercial or financial relationships that could be construed as a potential conflict of interest.

## Publisher's Note

All claims expressed in this article are solely those of the authors and do not necessarily represent those of their affiliated organizations, or those of the publisher, the editors and the reviewers. Any product that may be evaluated in this article, or claim that may be made by its manufacturer, is not guaranteed or endorsed by the publisher.

## Appendix

**Summary of Searches**

**1. Google Scholar**

“Cultural presentations of BPD”

**28,700 results**

**1 article** (Ronningstam et al., 2018)

**2. Embase**

“borderline personality disorder”:ti

AND

cultur^*^ OR race OR racial OR ethnic OR ethnicity OR ‘cross cultural' Or sociocultural OR immigrant^*^ OR immigration^*^ OR ‘socioeconomic status' OR social OR spiritual^*^ OR religious OR religion^*^

**1,044 results**

**1 article** (Haliczer et al., 2019)

**3. PubMed Search**

(suicide[ti] AND (pattern^*^[ti] OR behavior^*^[ti] OR behaviour^*^)) OR “parasuicidal”[ti] OR “self harm”[ti] OR “self injurious behavior^*^”[ti] OR “self-injurious behaviour^*^”[ti] OR “suicidal behavior^*^”[ti] OR “suicidal behaviour^*^”[ti] OR “suicide ideation”[ti] OR “suicidal ideation”[ti]

AND

(“North America” OR Canada OR Africa OR Asia OR India OR Europe OR China OR Japan OR Korea OR Taiwan OR Russia OR “South America” OR Eastern OR western OR race OR ethnicity OR origin OR “geographic region” OR “countries of origin” OR “country of origin” OR immigrant^*^ OR refugee^*^ OR Indigenous OR “First Nations” OR “Alaska Native” OR “Native American” OR latin^*^ OR Hispanic OR “African American” OR black)

AND

(sociocultural[TIAB] OR cultural[TIAB] OR psychology[TIAB] OR psychological[TIAB] OR “psychology” [Subheading])

**2,934 results**

**1 article** (Chowdhury et al., 2013)

**4. PubMed Main Search**

(borderline[tiab] OR “borderline disorder”[tiab] OR “borderline personality disorder”[tiab]) AND ((suicide[ti] AND (pattern^*^[ti] OR behavior[ti])) OR “parasuicidal”[ti] OR “self harm”[ti] OR “self injurious behaviors”[ti] OR “suicidal behavior^*^”[ti] OR “suicidal behavior^*^”[ti] OR “suicide ideation”[ti] OR “suicidal ideation”[ti]) AND (“North America” OR Canada OR Africa OR Asia OR India OR Europe OR China OR Japan OR Korea OR Taiwan OR Russia OR “South America” OR Eastern OR western OR race OR ethnicity OR origin OR “geographic region” OR “countries of origin” OR “country of origin” OR immigrant^*^ OR refugee^*^ OR Indigenous OR “First Nations” OR “Alaska Native” OR “Native American” OR latin^*^ OR Hispanic OR “African American” OR black)

**66 results**

**12 articles** (Jacobson et al., 2008; Nath et al., 2008; Holm and Severinsson, 2010; Wong et al., 2010; Cerutti et al., 2011, 2012; Gratz et al., 2012; Del Bello et al., 2015; Hu et al., 2016; Hayashi et al., 2017; Takeuchi et al., 2018; Chartrand et al., 2020)

**5. PubMed Search**

Self harm behavior prevalence in borderline personalitydisorder

**428 results**

**2 articles** (Bježančević et al., 2019; Levine et al., 2020)

**6. Reviewed the references of the article: “Prevalence of Borderline Personality Disorder and Its Clinical Correlates in Chinese Patients with Recent Deliberate Self-Harm”**

**4 articles** (Paris, 1996; Pinto et al., 2000; Söderberg, 2001; Wang et al., 2012)
